# Characterization of corn, cassava, and commercial flours: Use of amylase‐rich flours of germinated corn and sweet potato in the reduction of the consistency of the gruels made from these flours—Influence on the nutritional and energy value

**DOI:** 10.1002/fsn3.902

**Published:** 2019-03-10

**Authors:** Stephano Tambo Tene, Julie Mathilde Klang, Serge Cyrille Ndomou Houketchang, Gires Teboukeu Boungo, Hilaire Macaire Womeni

**Affiliations:** ^1^ Research Unit of Biochemistry, Medicinal Plants, Food Sciences and Nutrition Department of Biochemistry Faculty of Science University of Dschang Dschang Cameroon

**Keywords:** amylase‐rich flours, cassava, characterization, consistency, corn, sweet potato

## Abstract

Malnutrition appears in weaning age and is usually due to weaning food which is of low nutritional value. This problem led us to investigate the study of the physicochemical and functional properties of cassava flours and corn flours, and the fluidification of the gruels made from these flours by germinated yellow corn and sweet white potato flours. To do this, the approximate chemical composition, physical and functional properties, and ability of amylase‐rich flours to digest the starch in order to reduce consistency were evaluated. From these analyses, it emerges that the chemical composition, and physical and functional properties are influenced by the nature and the treatment undergone by the flours. It appears that the amylase‐rich flours that we used at a concentration of 1%–3% during the preparation of the gruels significantly reduced their consistencies. Given their strong liquefying power, this reduction was more marked with germinated corn flour where 1% permits to obtain desired consistency with 21.50 g of DM of bitter cassava flour, thereby multiplying the energy density and nutritional value of this flour by 5.18. It also appears that the action of flours rich in amylases was depending on the concentration, the nature of the flour, its composition, and the treatment undergone. In view of all these results, we can therefore consider the formulation of a weaning food with the consistency, and energy and nutritional value necessary for the proper growth of children.

## 1 INTRODUCTION

In tropical countries such as Cameroon, cereals, roots, and tubers are considered essential components of every meal and are introduced very early in children's complementary diets (FAO, 2014). These complementary foods are introduced into the young child's diet in order to vary his diet and the flavor of what he eats, to teach him to chew, grind, and swallow food and to accustom him to the flavors of home foods (Shipra, Neerubala, Shikha, & Shami, 2015). But despite the importance and high local productivity of the products used in the formulation of infant cereals, most of the infant meals offered in Cameroon are imported products at almost inaccessible prices for most families. To overcome this inaccessibility, they themselves produce complementary foods for their babies. However, failure to control key steps in the food processing process can impact on the nutritional value of food (macronutrients and micronutrients) and incorporate a risk of malnutrition in growing children (Gobson & Hotz, 2007). This infant malnutrition occurs mainly between the ages of 6 and 24 months, normally corresponding to the end of exclusive breastfeeding and the introduction of solid foods (De Benoist, 1995). In Cameroon, 33% of children under five suffer from malnutrition, more than half (18.4%) of which is severe (INS, 2014). This malnutrition is due to the fact that the infant gruels prepared daily from cereal flours (corn, e.g.), roots (cassava), and tubers (potato, sweet potato) by the parents are of low energy and nutritive density because they use low concentrations in dry matter of flours (5–10 g of MS). This low concentration of dry matter used during cooking of the spray liquid is due to the starch which swells during cooking and increases the consistency of the spray liquid. To give the gruels a sufficiently fluid consistency acceptable to the child's digestive system (30 ml/Kg body weight), mothers limit the proportion of flour to water and prepare gruels, which affects the energy and nutritional density of these (WHO, 1998). However, according to WHO and UNICEF, weaning gruels must have flow rates between 100 and 160 mm/30 s for a dry matter concentration of at least 30% (Zannou‐Tchoko, Ahui‐bitty, Kouame, Kouame, & Dally, 2011). Therefore, the most effective solution to increase the energy intake of children seems to be the implementation of treatments that reduce the consistency of gruels with high concentration in dry matter. One of these treatments is the depolymerization of starch which can be obtained by enzymatic hydrolysis. This hydrolysis is possible thanks to the use of flours or amylase extracts. Yadang, Mbome Lape, and Ndjouenkeu (2013) have demonstrated the increased capacity of unfermented sweet potato flour to reduce the viscosity of porridges made from fermented potato flour. In the same vein, Trèche (1999) demonstrates that the preparation of gruels of improved energy density can be done by using exogenous sources of amylases such as germinated cereal flours or amylase‐rich flours (ARF) which are the resultant of the germination of cereals such as rice (Singhavanich, Jittinandana, Kriengsinyos, & Dhanamitta, 1999), sorghum (Usha, Lakshmi, & Ranjani, 2010), barley (Elenga, Massamba, & Silou, 2012), and corn (Sodipo & Fashakin, 2011). In addition, this treatment will reduce the consistency and increase the nutritional value and energy density of weaning gruels. This work therefore aims to improve the fluidity, nutritional value, and energy density of gruels made from corn (Atp), cassava (bitter cultivar), and commercial flour by adding flour rich in amylases (germinated yellow corn and white sweet potato flours).

## 2 MATERIAL AND METHODS

### 2.1 Plant material

The plant material consists of seeds of Atp variety corn (yellow), cassava roots (bitter cultivar), tubers of local sweet potato variety (white) and commercial flour. The choice of these foods is justified by their common uses in the formulation of infant flours.

### 2.2 Methods

Supply of food raw material: Dry corn seeds, cassava roots, and potato tubers were purchased from the Dschang and Santchou IRAD stations, respectively. They were used to produce the various flours.

### 2.3 Flour production

Corn seeds obtained at IRAD were separated into three batches. The first lot was used for the production of sprouted corn flour and the other two batches for the production of corn flour. Indeed, for the production of germinated corn flours, the previously sorted corn seeds were washed and soaked for 48 hr. They were then removed from the water, spread out, and mixed with ashes and then for 96 hr, they were left in the shade on a cloth that we watered every day (02 watered/day) until the germination process was well underway and the roots appeared. Afterward, we proceeded to drying in a “Venticell” oven at a temperature of 50 ± 0.5°C for 45 hr. The seeds were then degerminated, ground, and sieved (Ø = 400 μm). The second batch was sorted, ground, and subsequently sieved (Ø = 400 μm). The third batch of each variety was also sorted, skinned, ground, and sieved (Ø = 400 μm). The flours obtained were then packed in plastic bags and stored in a desiccator. The cassava roots obtained were transformed into flour according to the method described by Bindzi (2012). This cassava flour was separated into two batches, one of which was roasted (120°C, 25 min). Local sweet potato tubers were washed and peeled. They were cut into slices (5–7 mm thick) using a stainless steel knife and dried for 24 hr in a “Venticell” oven set at 45 ± 0.5°C. The dried samples were then ground using a “Moulinex,” and the flour obtained was sieved (400 μm). The various corn flours, cassava flours, and commercial flour purchased on the market were subsequently characterized. Amylase‐rich flours were incorporated at 60°C during the preparation of the gruels.

### 2.4 Methods of analysis

**Figure 1:**
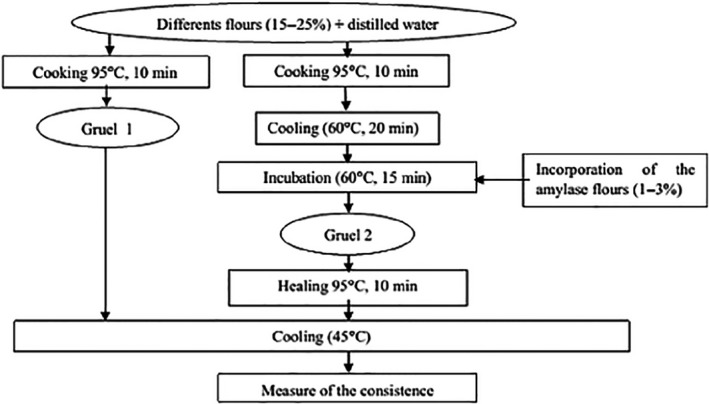
Diagram of preparation of gruels

The water content of each flour was obtained after drying in the oven at 105°C until constant weight and the dry matter determined after subtraction of the water content. Leach and Schoch (1959) method was used to determine water retention capacity. The mass density was determined using the method described by Okaka, Okorie, and Ozo (1991). pH, fibers, protein, ash, mineral (Ca, Cu, Fe, Mg, Na, P, and K), and fat and carbohydrate contents were determined according to the method described by the AOAC (1990), starch content according to the method described by Jarvis and Walker (1993). The method described by Fischer and Stein (1961) was used to measure reducing sugars. The titratable acidity was performed according to the method described by AFNOR (1982). The method described by Reddy, Manju, and Love (1999) was used to measure phytates in different flours. The cyanogenic glycoside content of the different flours was evaluated according to the method described by Makkar, Siddhuraju, and Becker (2007). The energy density was determined by a precise method by combining all the ingredients providing energy and using the coefficients of Atwater and Benedict (1899). DE = (G × 4) + (P × 4) + (L × 9). After the characterization of the flours, these were used for the preparation of the gruels with the introduction of amylase‐rich flours after cooking (Figure 1). Flours in concentrations ranging from 15 to 25 g MS were used for amylase‐rich flour concentrations ranging from 1% to 3%. Flow velocities of the different gruels were subsequently measured using a Bostwick consistometer (Bookwalter, Peplinski, & Pfeifer, 1968).

### 2.5 Statistical analysis

The results of the analyses carried out were expressed as averages plus or minus deviations. The means were analyzed by the ANOVA test at the 5% probability threshold, and the Duncan test was used to compare the means. The graphs were drawn using Excel 2013 software. A correlation matrix and principal component analysis (PCA) between the physicochemical, functional, and consistency properties of the gruels were also performed using SPSS software version 20.0 and XLSTAT 2016.

## 3 RESULTS AND DISCUSSION

### 3.1 Proximate chemical and mineral composition of the different flours

Table 1 shows the proximate overall chemical composition of the different flours used in this work. It was found that roasted bitter cassava flour had the lowest water content compared to commercial flour. Note that this parameter varies with the treatment applied to samples (*p *˂ 0.05). Except commercial flour, the water content values of flour are similar to those obtained by Ndangui (2015), on sweet potato flour which recommends a water content of <14% for a good conservation of flour. The protein content varies from 7.66% for commercial flour to 1.14% for roasted bitter cassava flour. The low protein content of roasted cassava flour can be explained by the fact that during roasting, the proteins were probably denatured or involved in the formation of melanoidins (Maillard reactions). This would justify the fact that this treatment lowers the protein content. However, there was no significant difference (*p* > 0.05) between the two cassava flours, which would mean that this treatment had not been extended. For corn flour, this protein content is not significantly different (*p* > 0.05). However, this level is lower than that found by Klang (2015), on corn flour of variety grown in Adamawa‐Cameroon, which was 10.2%, which is explained by the variety of corn, the locality where it was harvested, the cultivation practices, and the chemical composition of the soil. The data also tend to support Sylvestre and Arreaudeau (1983), who demonstrated that roots and tubers were not preferential sources of protein. In terms of fat content, yellow corn flour had the highest fat content (7.52%) and bitter cassava flour the lowest (0.63%). The significant difference (*p < *0.05) observed within the corn samples would be the consequence of the destruction of the germ, place of storage of lipids during the dehulling. In the case of cassava, this result obtained does not corroborate those of Fana, Shimelis, and Abrehet (2015), who demonstrate that during dried bleaching of potato, fatty acids are losing by evaporation. The carbohydrate content of each flour is significantly higher than other nutrients with values between 82.83% and 96.95% for yellow corn flour and bitter cassava flour, respectively. The significant difference (*p < *0.05) observed would be due to the treatments applied to the samples. The values obtained are similar than those of Belitz, Grosch, and Schieberle (2009), who worked on corn flour from a variety grown in Germany and found a total carbohydrate content of 96.1%. The dietary fiber content in the various flours is lower (*p < *0.05) than those found by Belitz et al. (2009), which was 9.8% in corn seeds. This low fiber content can be explained by climatic conditions, cultivation practices, and treatments applied. The ash content which is related to the extraction rate and mineralization of the ground grains differs significantly (*p < *0.05) within the samples. This difference would be due to the nature of the samples and the treatments applied. The lowest value of ash in roasted cassava is due to the sensitivity of specific minerals to temperature at prolonged time during roasting (Malomo, Jimoh, Adekoyeni, Soyebi, & Alamu, 2013). Chemical analysis of the starch content shows that it varies from 46.30% for yellow corn flour to 66.02% for bitter cassava flour. The significant differences (*p < *0.05) in content between husked and unpeeled corn flour can be explained by the fact that husking affected the germ where the starch was stored. Moreover, there is no significant difference (*p* ˃ 0.05) between the different cassava samples which would mean that the roasting was not pushed until browning and that we only had a pregelatinization of the starch without breaking the bonds that keep the molecule stable. Concerning amylose and amylopectin, we have observed that the treatment and the nature of the flours do not affect these parameters. The content of reducing sugars varies from 3.75% for commercial flour to 0.28% for dehulled yellow corn flour. It is observed that these levels are very low and this could be attributed to the accumulation of starch (complex carbohydrates) during maturation. The analyses carried out showed the existence in very small quantities of phytates in corn and commercial flours and an absence in cassava samples. These results could be due to crop conditions and the degradation of these compounds by phytases during plant development as reported by Medoua, Mbome Lape, Agbor‐Egbe, and Mbofung (2005), who observed a reduction in the phytates content of white yam tubers as maturation time evolved. The content of cyanogenetic compounds in cassava flours is higher than that found by Bindzi (2012), which was 0.37 mg HCN equivalent/100 g DM in cassava flour of a variety of the cultivated in the Central Cameroon region. This could be due to the drying method. Indeed, Bindzi (2012) demonstrated that drying cassava slice in the oven did not allow a better evaporation of cyanide from cassava compared to drying in the sun. Moreover, these results are not in agreement with those of Onimawo and Akpovwo (2006), who showed that during roasting at 100°C of pigeon pea, there was a significant decrease in cyanide contents (from 68 to 23 mg/100 g). Overall, the levels of cyanogenetic compounds found in flours comply with the FAO/WHO standard (maximum 10 mg HCN/Kg product equivalent). The calorific energy that gives us information on the energy intake of each flour studied varies from 424.72 Kcal for yellow corn flour to 398.95 Kcal for bitter cassava flour. These results show that this parameter is significantly affected (*p < *0.05) by the nature, nutritional composition, and treatment of the sample. These results are higher than those found by Ngouadjeu (2015), on some potato varieties from two production zones in Menoua (West‐Cameroon) whose values were 329.18 Kcal for the Tselefou Ft variety and 294.02 Kcal for the Sipiera Bf variety.

**Table 1:** Proximate chemical composition of the different flours

Flours	YCF	DYCF	BCF	RBCF	CF
Moisture (%)	13.29 ± 0.36^b^	12.48 ± 0.10^b^	12.55 ± 0.40^b^	10.12 ± 0.41^c^	14.98 ± 0.70^a^
Proteins (% of DM)	6.43 ± 0.31^b^	6.82 ± 0.30^b^	1.37 ± 0.02^c^	1.14 ± 0.15^c^	7.66 ± 0.18^a^
Lipids (% of DM)	7.52 ± 0.03^a^	4.23 ± 0.16^b^	0.63 ± 0.13^e^	1.72 ± 0.15^d^	3.41 ± 0.07^c^
Total carbohydrates (% of DM)	82.83 ± 0.13^c^	84.80 ± 0.40^bc^	96.95 ± 0.44^a^	95.99 ± 0.37^a^	87.13 ± 0.11^b^
Fibers (% of DM)	1.02 ± 0.00^a^	0.00 ± 0.00^b^	0.00 ± 0.00^b^	0.00 ± 0.00^b^	0.00 ± 0.00^b^
Ash (% of DM)	2.20 ± 0.14^b^	4.15 ± 0.07^a^	1.05 ± 0.21^d^	1.15 ± 0.07^d^	1.80 ± 0.00^c^
Starch (% of DM)	57.63 ± 0.76^a^	46.30 ± 3.36^b^	66.02 ± 9.14^a^	59.67 ± 3.00^a^	60.70 ± 5.20^a^
Amylose (%)	37.67 ± 3.81^b^	40.46 ± 1.16^a^	38.90 ± 1.86^ab^	37.83 ± 1.93^b^	42.73 ± 4.13^a^
Amylopectin (%)	62.33 ± 3.15^a^	59.54 ± 2.43^ab^	61.10 ± 7.29^a^	62.17 ± 3.23^a^	57.27 ± 4.30^b^
Reducing sugar (% of DM)	0.61 ± 0.40^b^	0.28 ± 0.13^c^	0.37 ± 0.04^c^	0.67 ± 0.07^b^	3.75 ± 0.20^a^
Cyanide (meq of HCN/% of DM)	0.00 ± 0.00^b^	0.00 ± 0.00^b^	0.73 ± 0.17^a^	0.65 ± 0.06^a^	0.00 ± 0.00^b^
Phytates (% of DM)	0.07 ± 0.001^a^	0.08 ± 0.005^a^	0.00 ± 0.000^c^	0.00 ± 0.000^c^	0.04 ± 0.000^b^
EV (Kcal/100 g of DM)	424.72 ± 0.40^a^	404.55 ± 1.10^bc^	398.95 ± 3.86^c^	404.00 ± 2.00^bc^	409.85 ± 0.35^b^

*Notes*

The values carrying the different letters (a, b, c…) in the same column meaningfully differ (*p* ˂ 0.05).

BCF: bitter cassava flour; CF: commercial flour; DYCF: dehulled yellow corn flour; EV: energy value; RBCF: roasted bitter cassava flour; YCF: yellow corn flour.

Flours are an abundant source of mineral salts. With regard to mineral composition (Table 2), potassium (K) is the most representative mineral in the various flours. These proportions vary from 569.00 mg/100 g of DM for bitter cassava flour to 124.95 mg/100 g of DM for husked yellow corn flour. Next comes phosphorus (P) whose proportions vary from 245.60 mg/100 g DM for yellow flour to 96.10 mg/100 g DM for commercial flour. Finally, minerals such as iron (Fe), calcium (Ca), and magnesium are the least represented in the different flours studied. In general, the content of these different ions is affected by the nature of the sample and the treatment applied (*p < *0.05). We observed that all mineral decreased with roasting probably due to the fact that thermal treatment denatures them. Recent report suggests that phytic acid contained in corn flour interferes with the availability of copper, iron, and potassium (Salunkhen, Chavan, Adsule, & Kadam, 1992). The significant differences observed in this mineral composition in the samples studied attest to the importance of ecological conditions and/or the influence of cultivation practices on the plant's mineralogy. Cultivation techniques and the chemical composition of the different cultivation sites could be factors in the mineral variability of the varieties of the same plant.

**Table 2:** Approximate mineral composition of the different flours

Flours	YCF	DYCF	BCF	RBCF	CF
Ca (mg/100 g of DM)	31.00 ± 0.30^a^	3.00 ± 0.00^d^	3.80 ± 0.28 ^cd^	4.50 ± 0.71^c^	8.20 ± 0.30^b^
P (mg/100 g of DM)	237.70 ± 1.55^b^	245.60 ± 1.30^a^	145.95 ± 0.64^c^	104.40 ± 0.60^d^	96.10 ± 0.42^e^
Na (mg/100 g of DM)	53.40 ± 0.00^a^	19.15 ± 0.50^e^	39.75 ± 0.50^c^	50.00 ± 0.85^b^	36.25 ± 1.20^d^
Mg (mg/100 g of DM)	17.11 ± 0.15^a^	1.21 ± 0.01^b^	1.95 ± 1.05^b^	1.21 ± 0.01^b^	1.21 ± 0.00^b^
Cu (mg/100 g of DM)	7.24 ± 0.06^c^	1.74 ± 0.03^e^	29.55 ± 0.10^a^	26.17 ± 0.10^b^	5.06 ± 0.02^d^
Fe (mg/100 g of DM)	7.30 ± 0.03^d^	7.90 ± 0.42 ^cd^	16.90 ± 0.85^a^	13.15 ± 0.92^b^	9.45 ± 0.92^c^
K (mg/100 g of DM)	349.45 ± 0.80^c^	124.95 ± 1.34^e^	569.00 ± 0.71^a^	549.00 ± 0.71^b^	134.90 ± 0.60^d^

*Notes*

The values carrying the different letters (a, b, c…) in the same column meaningfully differ (*p* ˂ 0.05).

BCF: bitter cassava flour; CF: commercial flour; DYCF: dehulled yellow corn flour; RBCF: roasted bitter cassava flour; YCF: yellow corn flour.

### 3.2 Ratio between the different minerals

Minerals represent the nutritive part of the flours. According to Kotowska and Wybieralski (1999), the nutritive value of edible vegetable parts is largely determined by the following ratios: K:Mg, Ca:Mg, Ca:P, and K:(Mg + Ca) (Table 3). Nutrient ratios present in Table 3 show that the mineral ratios were influenced by the nature of the flours and the treatment. According to Majkowska‐Gadomska and Wierzbicka (2008), the optimal Ca:Mg ratio should approximate 3, and the Ca:P ratio should be within the 1.2–2.2 range. The difference between this and our results can be explained by the nature of the flours, the treatment, and the culture conditions. A higher ratio is indicative of magnesium or phosphorus deficiency. All analyzed flours showed a higher potassium‐to‐magnesium ratio and the same for potassium‐to‐total magnesium and calcium ion ratio. According to Radkowski, Grygierzec, and Soek‐Podwika (2005), the optimal ratios K:Mg are 6 and K:(Mg + Ca) are 1.6–2.2. The difference observed can be due to fertilized and cultural conditions. Highest K:Mg ratio was noted in roasted bitter corn flour and the lowest in yellow corn flour.

**Table 3:** Ratio between the different minerals

Flours	YCF	DYCF	BCF	RBCF	CF
Ca:Mg	1.81 ± 0.03^c^	2.46 ± 0.00^bc^	2.32 ± 1.37^bc^	3.96 ± 0.57^b^	6.75 ± 0.23^a^
Ca:P	0.13 ± 0.00^a^	0.01 ± 0.00^c^	0.03 ± 0.00^d^	0.04 ± 0.01^c^	0.08 ± 0.00^b^
K:Mg	20.42 ± 0.22^a^	102.62 ± 0.80^bc^	254.16 ± 11.47^b^	450.92 ± 0.73^a^	111.02 ± 0.46^bc^
K:(Mg + Ca)	7.26 ± 0.20^d^	20.63 ± 0.30^c^	74.51 ± 9.91^b^	96.76 ± 11.90^a^	14.33 ± 0.37 ^cd^

*Notes*

The values carrying the different letters (a, b, c…) in the same column meaningfully differ (*p* ˂ 0.05).

BCF: bitter cassava flour; CF: commercial flour; DYCF: dehulled yellow corn flour; RBCF: roasted bitter cassava flour; YCF: yellow corn flour.

### 3.3 Functional properties of the different flours

**Figure 2:**
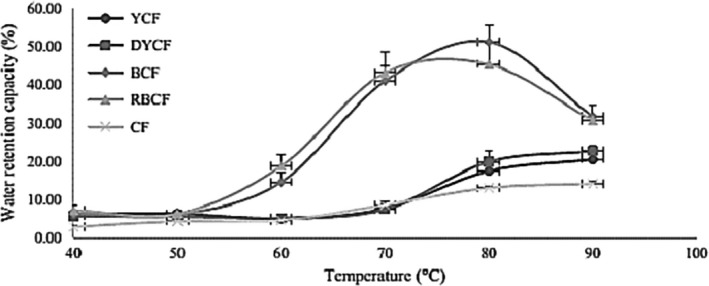
Water retention capacity of the different flours. BCF: bitter cassava flour; CF: commercial flour; DYCF: dehulled yellow corn flour; RBCF: roasted bitter cassava flour; YCF: yellow corn flour

Figure 2 shows the water retention capacity of the different flours and shows that the amount of water retained increases with temperature for the different flours with high water retention between 70 and 80°C for maize and commercial flours. This increase is justified by the fact that at high temperatures, the hydrogen bound stabilizing the semicrystalline structure of the starch open rupture and are replaced by water molecules and we are witnessing a release of the constituents of low molecular weight (amylose, intermediate material) of the granule (Tester & Karkalas, 1996). In addition, there is a better retention of undehulled corn flour, which would be the consequence of the elimination of fibers contained in the films and which would be a factor limiting water retention by the starch. The dehulling eliminates the phytates, which makes the phosphorus complex and thus limiting their capacities to bind water. For cassava flours, the high water retention is between 60 and 70°C with a peak at 80°C followed by a water retention fall between 80 and 90°C. These results are in agreement with those of Nuwamanya, Baguma, Emmambux, Taylor, and Rubaihayo (2010), who demonstrated that the gelatinization temperature of cassava starch is between 60 and 70°C and that after 80°C of heating of the latter, there was a burst. The burst at 80°C is due to bursting of starch granules.

**Figure 3:**
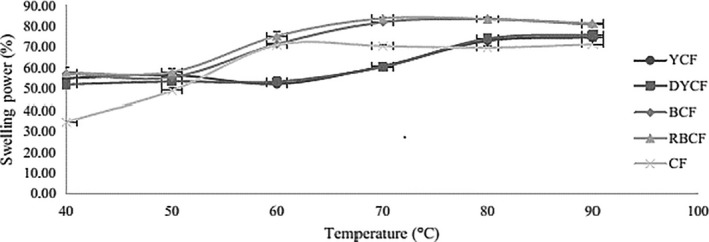
Swelling rate of the different flours. BCF: bitter cassava flour; CF: commercial flour; DYCF: dehulled yellow corn flour; RBCF: roasted bitter cassava flour; YCF: yellow corn flour

As far as the swelling rate is concerned, we are witnessing the same phenomena as with water retention capacity. The cassava flours have higher swelling power due to lower content in protein. Protein reduces the establishment of bound between starch and water (Figure 3).

### 3.4 Physical properties (MD and pH), WAC, titratable acidity, and SI of different flours

The physical properties of flours (mass density and pH) are shown in Table 4. The mass density varies from 0.47 g/ml for bitter cassava flour to 0.91 g/ml for decoupled yellow corn flour. There is a significant difference (*p < *0.05) between cassava and corn flours for this parameter due to the particle size of the samples (Adebowale, Sanni, & Oladapo, 2008) and the nutrient composition. These values are higher than those for corn flour obtained by Klang (2015), which was 0.55 g/ml. This difference is due to the difference of variety, treatment applied to it, and drying method used. Low densities (<0.5 g/ml) are recommended for the preparation of infant flours. The pH has an influence on the water retention capacity of flours and their rate of demotion. It is also an important parameter in the production of high viscosity starch gels. In our study, its value varies from 6.44 for dehulled yellow corn flour to 5.55 for roasted bitter cassava flour. There is a significant difference (*p < *0.05) in pH values between corn and cassava samples. This would be explained by the fact that the cassava samples were fermented and during this process there was synthesis of organic acids leading to a reduction in pH. The pH range obtained is the same with that of Fana et al. (2015). The titratable acidity which indicates the free acidity of the flours varies from 1.92 ml eq NaOH/100 g of DM for commercial flour to 3.92 ml eq NaOH/100 g of DM for roasted bitter cassava flour. As the results show, it is little affected by the treatment applied and the nature of the sample. The water absorption capacity as presented in Table 3 depends on the availability of hydroxyl groups that bind water molecules (Elenga et al., 2012). It should be noted that there is no significant difference (*p* ˃ 0.05) between the different cassava and corn samples and this could be explained by weak intermolecular hydrogen bonds and/or more water molecule binding sites. Regarding the solubility index, it is also noted that there is no significant difference (*p* ˃ 0.05) between the different samples of corn and cassava but rather between these samples and commercial flour. This would be explained by the composition of the commercial flour which would be rich in soluble compound. The value obtained is greater than that of Abioye, Ade‐Omowaye, Babarinde, and Adesigbin (2011), obtained in plantain flour. The difference observed is due to the nature of the sample, the chemical composition, the drying mode, and the treatment applied.

**Table 4:** Water absorption capacity, mass density (MD), pH, and titratable acidity of flours

Flours	YCF	BCF	DYCF	RBCF	CF
MD (g/ml)	0.84 ± 0.02^a^	0.47 ± 0.10^b^	0.91 ± 0.03^a^	0.55 ± 0.02^b^	0.83 ± 0.01^a^
pH	5.98 ± 0.13^b^	5.80 ± 0.02^c^	6.44 ± 0.03^a^	5.55 ± 0.06^d^	6.06 ± 0.01^b^
Titratable acidity (ml eq NaOH/100 g of DM)	3.75 ± 0.25^a^	3.74 ± 0.00^a^	2.74 ± 0.25^b^	3.92 ± 0.25^a^	1.92 ± 0.29^c^
WAC (%)	60.16 ± 0.40^b^	60.94 ± 0.43^b^	59.81 ± 0.20^b^	61.68 ± 0.45^b^	74.91 ± 11.53^a^
Solubility index (%)	16.39 ± 2.94^b^	15.19 ± 2.48^b^	15.42 ± 1.57^b^	12.11 ± 2.36^b^	56.86 ± 5.65^a^

*Notes*

The values carrying the different letters (a, b, c…) in the same column meaningfully differ (*p* ˂ 0.05).

BCF: bitter cassava flour; CF: commercial flour; DYCF: dehulled yellow corn flour; RBCF: roasted bitter cassava flour; WAC: water absorption capacity; YCF: yellow corn flour.

### 3.5 Use of amylase‐rich flours in reducing the consistency of gruels

#### 3.5.1 Evolution of gruel flow velocities without the use of amylase‐rich flours

**Figure 4:**
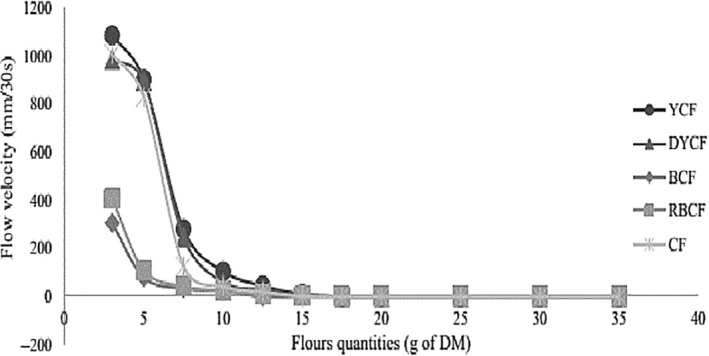
Evolution of the flow velocity of different flours in function of the concentration of substrate and in the absence of amylase flours. BCF: bitter cassava flour; CF: commercial flour; DYCF: dehulled yellow corn flour; RBCF: roasted bitter cassava flour; YCF: yellow corn flour

Figure 4 shows the evolution of the flow velocities of the gruels in the absence of amylase‐rich flours. Flow velocities decrease to 0.00 mm/30 s as the concentration of DM in the gruel increases. This is due to the fact that with the rise in temperature and dry matter of the flours, the starch granules swell due to the retention of water; this therefore gives the gruels a viscous character leading therefore to the reduction of the flow velocity as observed. The initial dry matter allows gruels reach a flow velocity between 100 and 160 mm/30 s, between 3% and 7.5% for cassava flour and 7.5% and 10% for corn flour. These gruels are therefore of low nutritional and energy values (<60 Kcal). This shows the interest of the use of amylase flours.

### 3.6 Effect of the use of amylase‐rich flours on the flow velocities of gruels with a 15% dry matter content

Table 5 shows that the incorporation of germinated yellow corn and white potato flours largely influences the flow velocities of the gruels. Thus, in the absence of amylase‐rich flours, flow velocities vary from 0.00 for cassava flours to 9.67 mm/30 s for yellow corn flour. It is noted that there is no significant difference (*p* < 0.05) within flours of the same nature but between flours of different nature. The significant difference (*p* < 0.05) observed within flours of different nature could be explained by the nutrient composition of these flours because the high proportion of proteins and lipids in corn flours would hinder the binding of water molecules by the starch during cooking, thereby preventing the gruels from gaining weight and consistency. This difference of flow rate can be also attributable to low starch content of corn (Table 1). This table also shows that the incorporation of amylase‐rich flours for the preparation of the various gruels makes it possible to multiply the flow velocities by 3 or even 5. Indeed, the incorporation of the germinated yellow corn flour makes it possible to obtain the flow velocities going from 189.33 for the yellow corn flour to 466.67 mm/30 s for the bitter cassava flour. Moreover, significant differences were observed (*p* < 0.05) between and within the different flours, which could be explained by their nutrient composition. Indeed, the strong activity of germinated yellow corn flour on cassava flours generally can be explained by the substrate specificity existing between the starch and the amylase of this flour; it would also be due to the fact that cassava flours are low in lipids and proteins which would hinder the establishment of amylase–substrate bonds and the hydrolysis of starch bonds leading to the reduction of its consistency. The use of white potato flour for the preparation of gruels has allowed us to obtain gruels with the flow velocities between 67.00 and 161.33 mm/30 s for bitter cassava and yellow corn flour, respectively. There was also a significant difference (*p* < 0.05) between the flow velocities of the different gruels cooking with white sweet potato flour. This would be explained by the nature of the amylase present in the flour and the composition of these flours. There is also a significant difference (*p* < 0.05) between the flow velocities of gruels supplemented with germinated corn flour and potato flour, which can be explained by the nature of the amylase contained in each amylase flour. Indeed, as demonstrated by Yadang et al. (2013), the potato is essentially rich in β‐amylase, and this enzyme compared to α‐amylase is more saccharogenic than amyloclastic hence the small reduction in consistency by the latter. This table also shows a significant difference (*p* < 0.05) between the flow rates of gruels with and without amylase flours, which would be the consequence of a rather significant degradation of starch by amylase content in amylase‐rich flours.

**Table 5:** Effect of the use of amylase flours (1%) on the flow velocity of gruels with a dry matter concentration of 15%

	F		
	WE	GYCF	WSPF
ARF			
YCF	9.67 ± 0.60^aC^	235.93 ± 1.44^cA^	161.33 ± 6.56^aB^
BCF	0.00 ± 0.00^bC^	466.67 ± 49.33^aA^	67.00 ± 7.00^cB^
DYCF	7.67 ± 2.52^aC^	189.33 ± 10.15^dA^	100.16 ± 9.08^bB^
RBCF	0.00 ± 0.00^bC^	358.67 ± 49.72^bA^	119.33 ± 15.63^bB^
CF	0.00 ± 0.00^bC^	218.33 ± 16.07^cA^	107.33 ± 3.05^bB^

*Notes*

The values carrying the different letters (a, b, c…) in the same column differ meaningfully (*p* ˂ 0.05). The values carrying the different letters (A, B, C…) in the same line differ meaningfully (*p* ˂ 0.05).

ARF: amylase‐rich flour; BCF: bitter cassava flour; DYCF: dehulled yellow corn flour; F: flours; GYCF: germinated yellow corn flour; RBCF: roasted bitter cassava flour; WE: without enzyme; WSPF: white sweet potato flour; YCF: yellow corn flour.

### 3.7 Effect of the use of amylase‐rich flours on the dry matter concentration of gruels with a flow velocity between 100 and 160 mm/30 s

Table 6 shows that the incorporation of germinated yellow corn and white sweet potato flours largely influences the dry matter concentration of the gruels. Thus, in the absence of amylase‐rich flours, the dry matter concentrations of flours vary from 4.15 for bitter cassava flour to 10.00% for yellow corn flour. The resulting gruels are low concentrations of dry matter. The incorporation of the germinated yellow corn flour makes it possible to prepare gruels having concentrations in DM ranging between 17.5% and 23%. In the presence of white sweet potato flour, dry matter concentrations ranging between 15% and 17.50% are obtained. We can therefore say that the low levels of germinated corn flour and sweet potato flour incorporated in the gruels of different flours make it possible to increase the DM concentrations of the flours. Similar results have also been obtained by Alvina, Vera, Pak, and Haraya (1990), who demonstrated that the incorporation of low doses of germinated corn flour (α‐amylases sources) in heavy and viscous gruels with initially no flow velocity results in an increase in flow velocity associated with an increase in dry matter. It also appears that the viscous character of corn, cassava, and commercial gruels disappears in the presence of germinated corn and sweet potato flours. This is due to the hydrolytic action of amylases which degrade large starch molecules into smaller molecules (maltodextrins, maltose...) whose reduced swelling capacity (Klang, 2015). The germinated corn flours, as well as those of sweet potato, thus make it possible to predigest the starch in order to make the gruels more digestible and easy to consume.

**Table 6:** Effect of incorporation of amylase‐rich flours on the dry matter concentration of gruels with a flow velocity between 100 and 160 mm/30 s

	F		
	WE	GYCF	WSPF
ARF			
YCF	10.00 ± 0.36	20.00 ± 0.36	16.50 ± 0.36
BCF	4.15 ± 0.40	21.50 ± 0.40	14.15 ± 0.40
DYCF	8.75 ± 0.10	17.50 ± 0.10	15.00 ± 0.10
RBCF	5.00 ± 0.41	20.00 ± 0.41	15.00 ± 0.41
CF	7.50 ± 0.69	20.00 ± 0.69	15.00 ± 0.69

*Note*

ARF: amylase‐rich flour; BCF: bitter cassava flour; DYCF: dehulled yellow corn flour; F: flours; GYCF: germinated yellow corn flour; RBCF: roasted bitter cassava flour; WE: without enzyme; WSPF: white sweet potato flour; YCF: yellow corn flour.

### 3.8 Evolution of the flow velocities of different gruels as a function of the concentrations of different amylase‐rich flours

**Figure 5:**
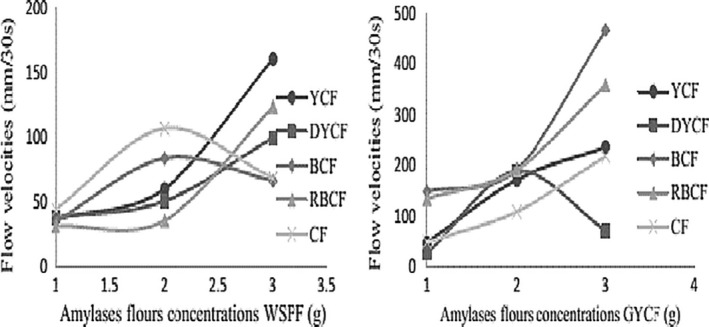
Evolution of gruel flow velocities in function of different amylase‐rich flour concentrations. BCF: bitter cassava flour; DYCF: dehulled yellow corn flour; GYCF: germinated yellow corn flour; RBCF: roasted bitter cassava flour; WSPF: white sweet potato flour; YCF: yellow corn flour

The evolution of the flow velocities of gruels in which the various amylase flours were added after cooking is given in Figure 5. This figure shows that the peak in the reduction of the consistency of cassava and commercial flours was observed with 2 g of sweet potato amylase‐rich flour followed by a decrease to 3 g. This could be explained by the composition of the different flours. Indeed, beyond 2 g, amylases that need water for their activity are in competition with the starch contained in these flours for mobility and their actions. Moreover, this could also mean that the starch contained in the amylase flour is in large proportion and has fixed the water leading to the increase in consistency. As far as the other flours are concerned, their flow velocities increase with the increase in the concentration of sweet potato flour. This means that the sweet potato amylases easily access the starch of these flours and therefore cause their hydrolysis leading to a reduction in consistency. This increase in the flow velocities is explained by a rather significant degradation of the starch. With regard to the action of corn flour rich in amylase, all the other gruels showed flow velocities proportional to the increase in the concentration of amylase flours, except for dehulled corn flour whose flow velocity decreased to a concentration of 3 g of amylase flour. We observe that the action of amylases in this flour rich in amylases depends on the treatment undergone by the flour as well as its composition. These results allow us to conclude that the incorporation of low doses of amylase flours (germinated corn and sweet potato) in heavy, viscous gruels with no initial flow velocity leads to the degradation of large starch molecules into smaller molecules (maltodextrins) with reduced swelling capacity.

### 3.9 Effect of the use of amylase‐rich flours on the nutritional value of different gruels with flow velocities between 100 and 160 mm/30 s

This Table 7 shows that the introduction of amylase sources during the preparation of gruels based on different flours implies an increase in nutritional value compared to gruels not supplemented. As a result, the multiplication of the nutritional value of the supplemented gruels not varies from 1.2 for the mixture of yellow corn flour + white sweet potato flour to 5.18 for the mixture of bitter cassava flour/germinated yellow corn flour. The table also shows the best combinations between each flour and the amylase‐rich flours to obtain the possible best nutritional value. These combinations are as follows: yellow corn flour/germinated yellow corn flour; bitter corn flour/germinated yellow corn flour; dehulled yellow corn flour/germinated yellow corn flour; roasted bitter corn flour/germinated yellow corn flour; and finally, commercial corn flour/germinated yellow corn flour. The results obtained with regard to the multiplication factor between amylase and nonamylase gruels are in line with those obtained by Zannou‐Tchoko et al. (2011), which showed that the introduction of 10% of germinated corn flour in gruels made from attiéké flour/soya and cassava flour/soya made it possible to obtain a flow velocity of 120 mm/30 s for a dry matter of 30.5% multiplying by 3.81 times the concentration in dry matter of the traditional gruel. Moreover, the low nutritional value conferred by the use of white potato flour during this cooking would be the consequence of its richness in starch because the flours used besides amylases also contain starch. Indeed, amylases are hydrolases that need water for their action and are therefore in competition with starch to fix it.

**Table 7:** Effect of incorporation of amylase‐rich flours on the nutritional value of different porridges with a flow rate between 100 and 160 mm/30 s

	F				
	WE	GYCF		WSPF	
		W	MF	W	MF
ARF					
YCF	10.00	20.00	2.00	16.50	1.65
BCF	4.15	21.50	5.18	17.50	3.41
DYCF	8.75	17.50	2.00	15.00	1.71
RBCF	5.00	20.00	4.00	15.00	3.00
CF	7.50	20.00	2.70	15.00	2.00

*Note*

ARF: amylase‐rich flour; BCF: bitter cassava flour; DYCF: dehulled yellow corn flour; F: flours; GYCF: germinated yellow corn flour; MF: multiplication factor; RBCF: roasted bitter cassava flour; W: with enzyme; WE: without enzyme; WSPF: white sweet potato flour; YCF: yellow corn flour.

### 3.10 Effect of the use of amylase‐rich flours (ARF) on the energy density of different gruels with a flow velocity between 100 and 160 mm/30 s

The influence of the use of the two amylase flours on the energy density of the gruels (Table 8) shows that in the absence of ARF, the gruels have low energy values. Indeed, these gruels have energy densities varying from 16.55 for bitter cassava flour to 42.47 Kcal for yellow corn flour. The significant differences (*p < *0.05) observed for this parameter between gruels without ARF can be explained by the composition and nature of the different flours. The introduction of ARF generally leads to an increase in energy density. Indeed, the incorporation of germinated yellow corn flour makes it possible to obtain energy densities going from 70.80 for the dehulled yellow corn flour to 91.75 Kcal for the bitter cassava flour. The significant differences (*p < *0.05) observed within the different flours can be explained by their nutrient composition and the ability of yellow corn flour to predigest starch in order to increase the quantity of flour and consequently the energy value. The incorporation of white sweet potato flour during the preparation of gruels has allowed us to obtain gruels with energy densities between 61.48 and 70.70 Kcal for commercial flour and roasted bitter cassava flour, respectively. In addition, a significant difference was observed (*p < *0.05) between the energy densities of gruels cooking with and without amylase‐rich flours which would show the ability of amylases contain in flours to increase the energy value of the gruels. Similar results were also obtained by Elenga, Massamba, Kobawila, Makosso, and Silou (2009), who demonstrated that the energy density of traditional gruels is around an average of 30–60 Kcal/100 ml of gruel and that such gruels are not able to effectively cover the nutritional needs of infants and young children in addition to breastmilk especially that the frequency of daily consumption of gruels is two times/day (Trêche, de Benoist, Benbourzid, & Delpeuch, 1995). These results therefore demonstrate that the incorporation of small quantities of germinated corn and sweet potato flour leads to an increase in energy density, as shown by the results of Zannou‐Tchoko et al. (2011) that an introduction of 15% of corn malt in attiéké flour + soybean gruels makes it possible to obtain an energy density of 126.1 Kcal/100 ml of gruel.

**Table 8:** Effect of incorporation of amylase‐rich flours on the energy density (in Kcal) of the different porridges with a flow velocity between 100 and 160 mm/30 s

	F		
	WE	GYCF	WSPF
ARF			
YCF	42.47 ± 0.03^aC^	84.94 ± 0.07^aA^	70.08 ± 0.06^aB^
BCF	16.55 ± 0.12^dC^	91.75 ± 1.37^aA^	69.81 ± 0.40^aB^
DYCF	39.00 ± 0.11^bC^	70.80 ± 0.25^dA^	60.70 ± 0.21^bB^
RBCF	20.20 ± 0.11^dC^	80.80 ± 0.06^bA^	70.70 ± 0.32^aB^
CF	30.74 ± 0.02^cC^	81.97 ± 0.42^bA^	61.48 ± 0.06^bB^

*Notes*

The values carrying the different letters (a, b, c…) in the same column differ meaningfully (*p* ˂ 0.05). The values carrying the different letters (A, B, C…) in the same line differ meaningfully (*p* ˂ 0.05).

ARF: amylase‐rich flour; BCF: bitter cassava flour; DYCF: dehulled yellow corn flour; F: flours; GYCF: germinated yellow corn flour; RBCF: roasted bitter cassava flour; WE: without enzyme; WSPF: white sweet potato flour; YCF: yellow corn flour.

### 3.11 Influence of physicochemical and functional properties on the ability of amylase‐rich flours to reduce the consistency of gruels: Pearson correlation matrix and principal component analysis (PCA)

Table 9 presents the correlation matrix between the physicochemical and functional properties and flow velocities due to different ARF. The correlations marked in bold and colored are significant (*p* < 0.01 and *p* < 0.05, respectively). This matrix shows that the variables are correlated in small groups. Hence, the interest of conducting an analysis is of main component in order to visualize the proximities and distances between these variables. The principal component analysis (PCA) allows us to map variables (starch, fat, fibers, protein, phosphorus, phytates, cyanide, water retention capacity, water absorption capacity, solubility index, Ca, Na, Mg, Cu, Fe, K, swelling rate, amylose, amylopectin, reducing sugars, ash, phenols, titratable acidity, pH, carbohydrates, mass density, and flow velocity due to GYCF and WSPF) according to their correlation and proximity.

**Table 9:** Contributions and square cosines of variables

	Contributions (%)			Square cosines (%)		
	F1	F2	F3	F1	F2	F3
Li	**5.186**	0.862	2.040	**0.887**	0.065	0.048
Pro	**5.783**	0.010	0.445	**0.989**	0.001	0.011
Ash	**2.895**	**6.703**	0.011	**0.495**	**0.505**	0.000
Car	**5.570**	0.634	0.001	**0.952**	0.048	0.000
Fib	0.000	0.000	0.000	0.000	0.000	0.000
Phe	0.018	**13.054**	0.591	0.003	**0.983**	0.014
Cyan	**5.820**	0.053	0.043	**0.995**	0.004	0.001
MD	**5.535**	0.595	0.376	**0.946**	0.045	0.009
WRC	**5.052**	1.789	0.064	**0.864**	0.135	0.002
WAC	1.767	**9.243**	0.081	0.302	**0.696**	0.002
SwP	**4.851**	2.033	0.740	**0.829**	0.153	0.018
Ca	1.088	**10.787**	0.078	0.186	**0.812**	0.002
Mg	2.395	0.003	**24.866**	0.410	0.000	**0.590**
K	**5.247**	0.131	3.919	**0.897**	0.010	0.093
Na	**2958**	4.994	4.982	**0.506**	0.376	0.118
Cu	**5.696**	0.331	0.051	**0.974**	0.025	0.001
Fe	**4.999**	0.523	4.460	**0.855**	0.039	0.106
P	0.549	**11.394**	2.030	0.094	**0.858**	0.088
pH	**4.007**	3.053	3.585	**0.685**	0.230	0.085
TA	**5.279**	1.108	0.590	**0.903**	0.083	0.014
Starch	0.611	**10.726**	3.708	0.104	**0.808**	0.088
AM	**4.586**	1.740	3.574	**0.784**	0.131	0.085
AMYP	**4.586**	1.740	3.574	**0.784**	0.131	0.085
RS	2.310	**8.036**	0.001	0.395	**0.605**	0.000
Phy	**4.734**	2.508	0.074	**0.809**	0.189	0.002
SI	2.310	**7.344**	0.927	0.425	**0.553**	0.022
GYCF	**5.276**	0.2499	3.336	**0.902**	0.019	0.079
WSPF	0.714	0.358	**35.853**	0.122	0.027	**0.851**

*Note*

AM: amylose; AMYP: amylopectin; Car: carbohydrates; Cyan: cyanide; Fib: fibers; GYCF: germinated yellow corn flour; Li: lipids; MD: mass density; Phe: phenols; Phy: phytates; Pro: proteins; RS: reducing sugar; SI: solublility index; SwP: swelling power; TA: titratable acidity; wac: water absorption capacity; wrc: water retention capacity; WSPF: white sweet potato flour.

The bold values in the table mean that these variables contribute significantly to the formation of these axes

**Figure 6:**
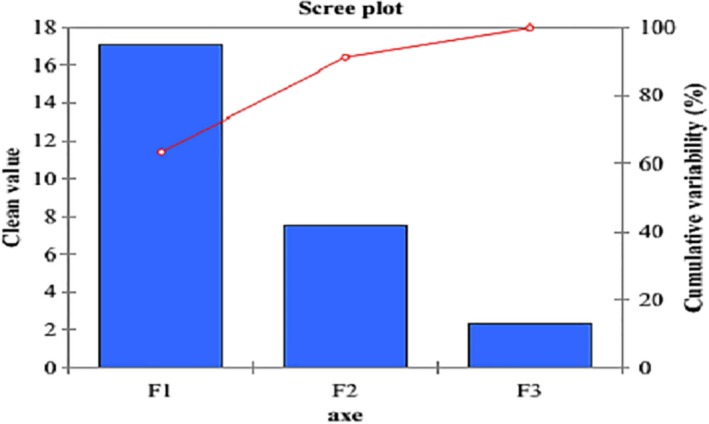
Characteristics of the main components

In the case of our analysis, three components F1, F2, and F3 explain 100% of the variations with respective contributions of 63.32%, 27.89%, and 8.79% (Figure 6).

**Figure 7:**
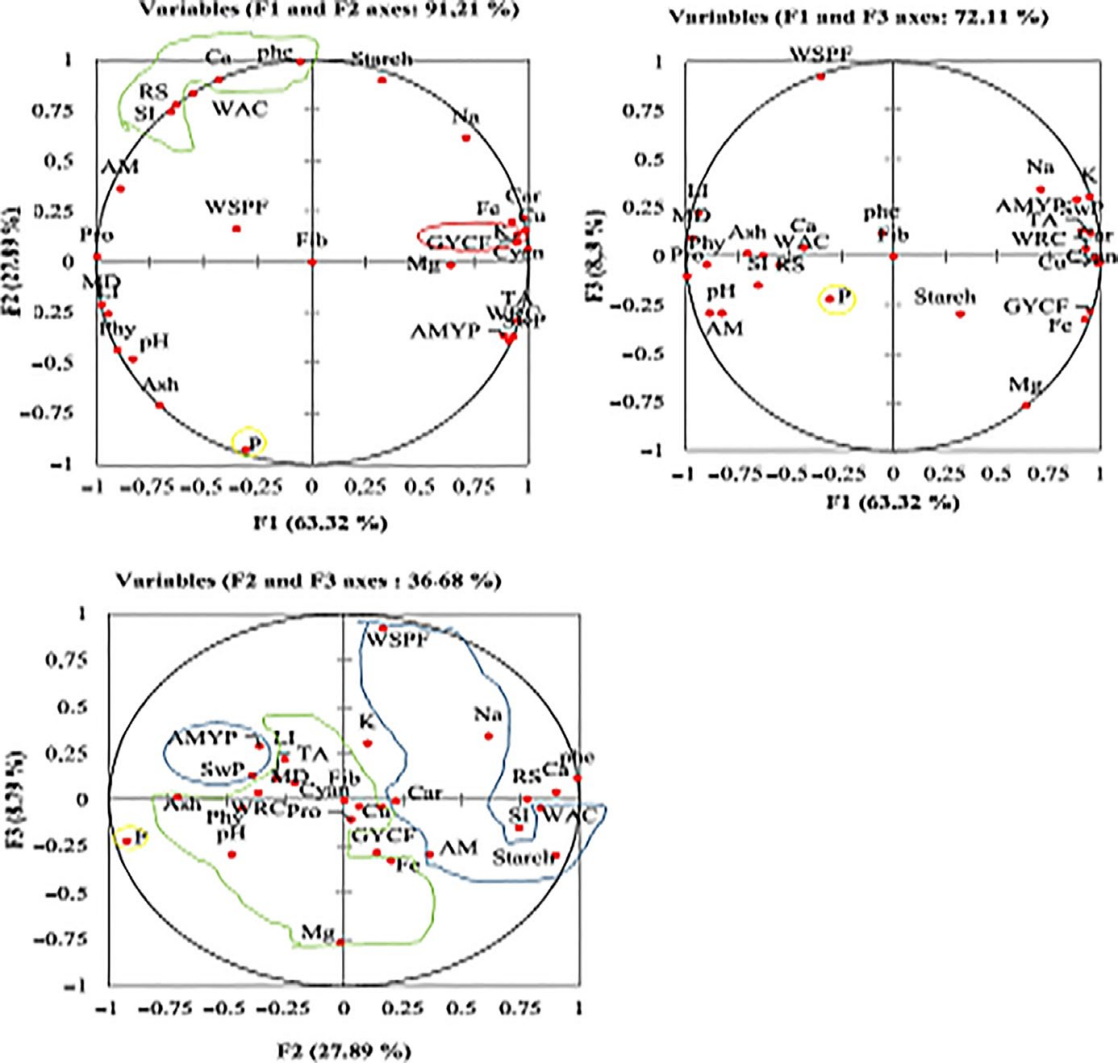
PCA diagrams of the relative variables to the physicochemical characteristic, functional properties, and flow velocities of the flours. Car: carbohydrates; Cyan: cyanide; Fib: fibers; GYCF: germinated yellow corn flour; Li: lipids; MD: mass density; Phe: phenols; Phy: phytates; Pro: proteins; RS: reducing sugar; SI: solublility index; SwP: swelling power; TA: titratable acidity; wac: water absorption capacity; wrc: water retention capacity; WSPF: white sweet potato flour

**Figure 8:**
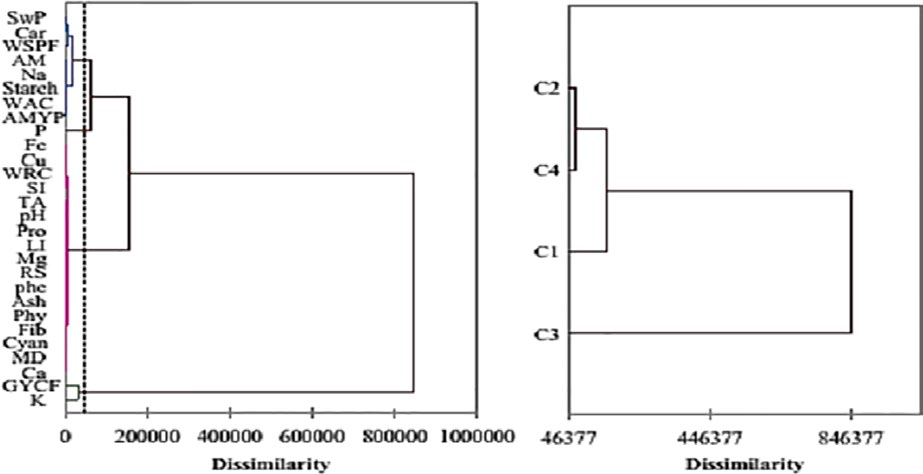
Dendogram of chemical composition, physical and functional properties, and flow velocities. AM: amylose; AMYP: amylopectin; Car: carbohydrates; Cyan: cyanide; Fib: fibers; GYCF: germinated yellow corn flour; Li: lipids; MD: mass density; Phe: phenols; Phy: phytates; Pro: proteins; RS: reducing sugar; SI: solublility index; SwP: swelling power; TA: titratable acidity; wac: water absorption capacity; wrc: water retention capacity; WSPF: white sweet potato flour

The PCA diagram of variables, also called variable correlation circles, and the variable dendogram allow us to visualize the grouping between these factors. This figure confirms once again the correlations but also the proximity between the different variables studied. These figures also show that the formation of these axes is dependent on the variables; this is how carbohydrates, lipids, proteins, cyanide, water retention capacity, swelling power, K, Na, Cu, Fe, pH, amylose, amylopectin, phytates, mass density, fibers, and flow velocities due to germinated yellow corn flour allow the formation of the F1 axis. The grouping of solubility index, reducing sugar, starch, P, Ca, and total phenols allows the formation of the F2 axis. Mg and flow velocities due to white sweet potato flour allow the formation of F3 axis. From these analyses, it emerges that the three axes make it possible to group the variables into four blocks according to PCA and dendogram (Figures 7 and 8).

The analysis of observations shows that three components F1, F2, and F3 explain 100% of the variations with respective contributions of 62.31%, 28.76%, and 8.96% (Table 10). These analyses also show that we have four active observations (DYCF, BCF, CF, and RBCF) and one complementary (DYCF). Among these observations, BCF, RBCF, and CF participating in the formation of axis F1; DYCF axis F2 and the complementary observation YCF F3 axis (Table 11).

**Table 10:** Contributions and square cosines of observations

	Contributions (%)			Square cosines (%)		
	F1	F2	F3	F1	F2	F3
DYCF	20.407	54.587	0.006	0.459	**0**.**540**	0.000
BCF	30.169	0.002	44.829	0.829	0.000	0.171
CF	29.537	44.868	0.595	0.598	0.400	0.002
RBCF	19.888	0.543	54.569	0.718	0.009	0.273
YCF				0.040	0.007	0.953

*Note*

BCF: bitter cassava flour; DYCF: dehulled yellow corn flour; RBCF: roasted bitter cassava flour; YCF: yellow corn flour.

The bold values in the table mean that these observations contribute significantly to the formation of these axes

**Table 11:** Pearson correlation coefficient (*r*) matrix between physicochemical and functional properties and flow velocities

Variables	Li	Pro	Ash	Car	Phe	Cyan	MD	WRC	WAC	SwP	Ca	Mg	K	Na	Cu	Fe	P	pH	TA	Starch	AM	AMYP	RS	Phy	SI	GWCF	YSPF
Li	**1**																										
Pro	**0.907**	**1**																									
Ash	**0.847**	*0.679*	**1**																								
Car	−**0.976**	−**0.964**	−**0.842**	**1**																							
Phe	−0.174	0.070	−*0.663*	0.162	**1**																						
Cyan	−**0.962**	−**0.987**	*−0.747*	**0.987**	0.003	**1**																					
MD	**0.991**	**0.952**	**0.836**	−**0.996**	−0.145	−**0.987**	**1**																				
WRC	*−0.773*	−**0.938**	−0.393	**0.827**	−0.411	**0.903**	−**0.823**	**1**																			
WAC	0.295	0.574	−0.207	−0.354	**0.852**	−0.494	0.354	−**0.819**	**1**																		
SwP	*−0.729*	−**0.938**	−0.361	**0.803**	−0.423	**0.886**	*−0.791*	**0.995**	−**0.833**	**1**																	
Ca	0.186	0.449	−0.336	−0.224	**0.923**	−0.375	0.233	*−0.730*	**0.987**	*−0.740*	**1**																
Mg	*−0.786*	−0.558	−0.451	*0.624*	−0.142	*0.662*	*−0.692*	0.571	−0.331	0.487	−0.323	**1**															
K	−**0.850**	−**0.970**	*−0.732*	**0.945**	0.082	**0.941**	−**0.914**	**0.856**	−0.451	**0.864**	−0.306	0.370	**1**														
Na	*−0.750*	*−0.726*	−**0.930**	**0.826**	*0.602*	*0.737*	*−0.789*	0.449	0.106	0.453	0.261	0.181	**0.839**	**1**													
Cu	−**0.977**	−**0.973**	−**0.807**	**0.998**	0.098	**0.995**	−**0.997**	**0.858**	−0.409	**0.832**	−0.285	*0.656*	**0.940**	*0.787*	**1**												
Fe	−**0.993**	−**0.881**	*−0.797*	**0.947**	0.107	**0.945**	−**0.972**	*0.774*	−0.328	*0.721*	−0.234	**0.838**	*0.796*	*0.687*	**0.955**	**1**											
P	0.476	0.302	**0.870**	−0.500	−**0.927**	−0.357	0.473	0.047	−0.595	0.054	*−0.712*	−0.013	−0.449	−**0.861**	−0.441	−0.396	**1**										
pH	**0.837**	**0.840**	**0.918**	−**0.911**	−0.464	−**0.846**	**0.879**	*−0.605*	0.068	*−0.605*	−0.088	−0.298	−**0.921**	−**0.983**	−**0.882**	*−0.765*	*0.762*	**1**									
TA	*−0.795*	−**0.965**	−0.461	**0.863**	−0.325	**0.926**	−**0.852**	**0.994**	*−0.768*	**0.994**	*−0.665*	0.522	**0.907**	0.539	**0.888**	*0.783*	0.058	*−0.682*	**1**								
Starch	−0.598	−0.267	−**0.871**	0.513	**0.838**	0.388	−0.533	−0.041	0.585	−0.097	*0.658*	0.420	0.305	*0.679*	0.471	0.574	−**0.866**	*−0.612*	0.012	**1**							
AM	*0.678*	**0.920**	0.361	*−0.784*	0.374	−**0.851**	*0.757*	−**0.967**	**0.802**	−**0.987**	*0.696*	−0.349	−**0.892**	−0.508	−**0.807**	*−0.652*	0.000	*0.644*	−**0.980**	0.126	**1**						
AMYP	*0.678*	−**0.920**	−0.361	*0.784*	−0.374	**0.851**	*−0.757*	**0.967**	−**0.802**	**0.987**	*−0.696*	0.349	**0.892**	0.508	**0.807**	*0.652*	0.000	*−0.644*	**0.980**	−0.126	−**1.000**	**1**					
RS	0.395	*0.645*	−0.110	−0.443	**0.807**	−0.578	0.447	−**0.869**	**0.994**	−**0.876**	**0.972**	−0.419	−0.516	0.032	−0.498	−0.428	−0.529	0.146	−**0.821**	0.494	**0.836**	−**0.836**	**1**				
Phy	**0.949**	**0.887**	**0.941**	−**0.973**	−0.386	−**0.923**	**0.963**	*−0.678*	0.134	*−0.655*	−0.005	−0.537	−**0.908**	−**0.921**	−**0.955**	−**0.904**	*0.687*	**0.965**	*−0.734*	*−0.669*	*0.652*	*−0.652*	0.207	**1**			
SI	0.392	*0.684*	−0.072	−0.473	*0.756*	−0.599	0.463	−**0.885**	**0.985**	−**0.904**	**0.945**	−0.315	−0.589	−0.059	−0.521	−0.407	−0.456	0.226	−**0.852**	0.502	**0.890**	−**0.890**	**0.987**	0.270	**1**		
GWCF	−**0.991**	−**0.912**	*−0.770*	**0.958**	0.050	**0.965**	−**0.979**	**0.821**	−0.395	*0.774*	−0.298	**0.822**	**0.827**	*0.663*	**0.969**	**0.997**	−0.356	*−0.770*	**0.829**	0.514	*−0.709*	*0.709*	−0.492	−**0.902**	−0.476	**1**	
YSPF	0.490	0.252	0.144	−0.309	0.291	−0.368	0.392	−0.549	0.288	−0.260	0.338	−**0.935**	−0.033	0.169	−0.351	−0.591	−0.242	−**0.059**	−0.270	−0.239	0.100	−0.100	0.352	0.204	0.213	−0.569	**1**

*Notes*

Italic values: *p* < 0.05; bold values: *p* < 0.01; plain values: nonsignificant.

AM: amylose; AMYP: amylopectin; Car: carbohydrates; Cyan: cyanide; Fib: fibers; GYCF: germinated yellow corn flour; Li: lipids; MD: mass density; Phe: phenols; Phy: phytates; Pro: proteins; RS: reducing sugar; SI: solublility index; SwP: swelling power; TA: titratable acidity; wac: water absorption capacity; wrc: water retention capacity; WSPF: white sweet potato flour.

**Figure 9:**
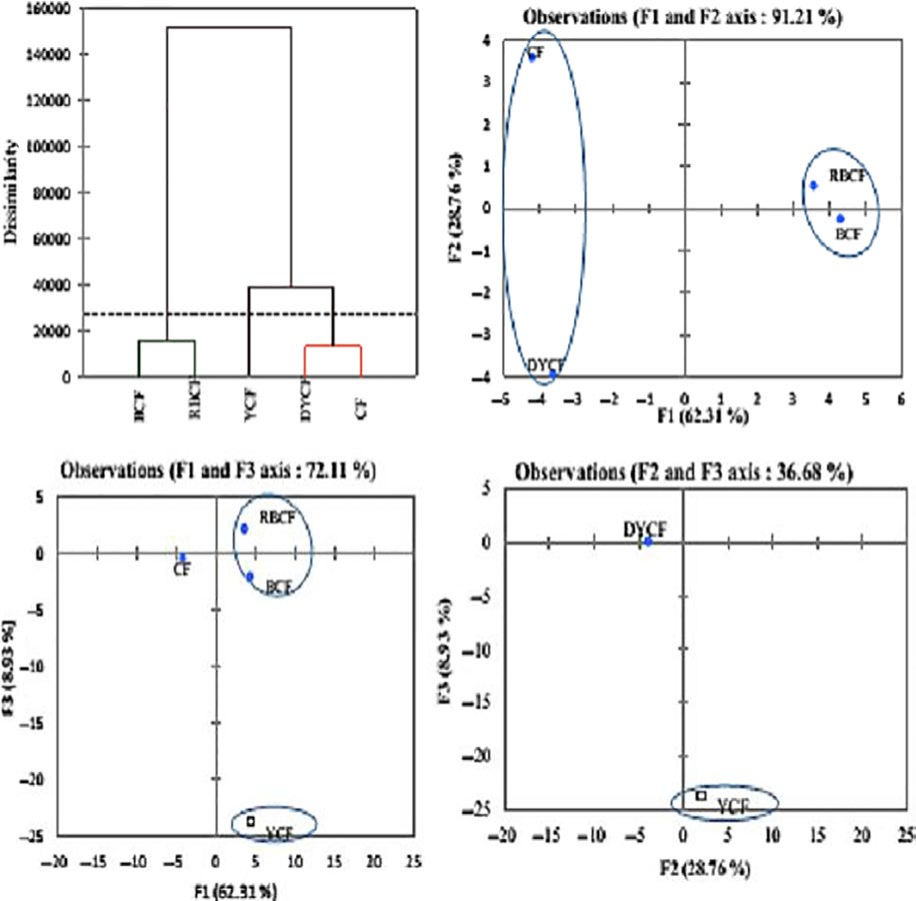
Flour dendogram and PCA pattern of observation flours. BCF: bitter cassava flour; DYCF: dehulled yellow corn flour; RBCF: roasted bitter cassava flour; YCF: yellow corn flour

We also subjected the observations to a hierarchical bottom‐up classification with a view to classifying the different classes and this allowed us to group them into three classes as shown in Figure 9.

Concerning the variables, the first grouping shows that germinated yellow corn flour and K are significantly and positively correlated (*r* = 0.827). This is explained by the fact that the amylases present in these flours require the presence of cofactors (ions) for their activity. The K would play this role at some concentration. These amylases are therefore metalloenzymes. These results corroborate those of Noman, Hoque, Sen, and Karim (2006), who demonstrated that the ions present in flours rich in amylases bind very strongly with these amylases and they are therefore necessary for activity and stability.

The second grouping is that between Ca, Mg, reducing sugars, proteins, pH, mass density, cyanide content, titratable acidity, solubility index, fibers, phytates, ashes, lipids, Fe, copper, phenols, and water retention capacity. This grouping shows that there is a strong negative correlation between phytates and the following ions: Fe (*r* = −0.904), Cu (*r* = −0.955), and Mg (*r* = −0.908). Phytates are antinutritional chelators of metals present in plants. Their presence in plants reduces the bioavailability of ions as demonstrated by Medoua et al. (2005). Another correlations are between proteins and phytates (*r* = 0.887). This can be explained by the fact that phytate molecule is negatively highly charged and this making it an excellent chelator who can form insoluble complexes with proteins leading to reduced digestibility (Wedad, Abdelhaleem, Abdelmoneim, & Elfadil, 2008). It also shows a significant and positive correlation between mass density and proteins (*r* = 0.952), lipids (*r* = 0.991), and pH (*r* = 0.879). Parameters without values in the table do not have significant effects (*p* < 0.05 and *p* < 0.01). Mass density is a parameter dependent on sample size and biochemical composition (particularly proteins) (Adebowale et al., 2008). Indeed, lipids and proteins are high molecular weight compounds and their high proportions in flours would give them a high mass density. The pH is characteristic of the acidity and basicity of flours. The high pH values are characteristic of the presence of macromolecules which would therefore increase the mass density. There is also a significant correlation between water retention capacity and Fe (*r* = 0.774), Cu (*r* = 0.858), Ca (*r* = −0.703), lipids (*r* = −0.773), cyanides (*r* = 0.903), proteins (*r* = −0.938), reducing sugars (*r* = −0.678), and mass density (*r* = −0.823). Ions such as Fe, Cu, and Mg are present on the surface of flours and more particularly starch. These ions allow the starch of the flours to establish bonds with the water molecules by electrostatic attractions and thus to retain this one. As for Ca, it prevents the binding of water molecules by phosphorous ions present on the surface of flour starches either by neutralizing the negative charge of these ions by establishing electrostatic bonds between them or by reducing the bioavailability of water by binding these molecules. For fixing water, proteins and lipids are found on the surface of starch granules and limit the binding of water molecules by the granule (Debet & Gidley, 2007). Cyanide is present in plants as cyanogenetic glycosides. The hydrolysis of this molecule during different treatments would release in addition to HCN a carbohydrate molecule capable of retaining water and thus increase this parameter. Indeed, proteins mask the starch molecule and more particularly these constituents thus reducing access to water or starch–water bonds. Zhang and Hamaker (1998) showed that the proteins present in sorghum flour reduced starch–water binding. It has also been found that the removal of gluten from wheat flour increases the water retention rate of its starch (Jenkins et al., 1987). Lipids are present in starch in the form bound to amylose (amylose‐lipids). They therefore reduce the degree of solubility of this molecule and the ability of these −OH groups to establish bonds with water (prevents amylose–water bonds). Reducing sugars are polysaccharide monomers, especially starch. They have a low swelling power and a low water retention capacity compared to these polysaccharides. Their presence in large proportion in a matrix would therefore lead to a reduction in this parameter. Finally, there is a strong correlation between the solubility index and proteins (*r* = 0.684), Ca (*r* = 0.945), phenols (*r* = 0.756), and reducing sugars (*r* = 0.987). Depending on the type of fatty acids and amino acids constituting the lipid and protein chain, respectively, this will influence their solubilities. Indeed, the richer the lipids are in phospholipids, the more soluble they are; the richer the proteins are in hydrophilic amino acids, the more soluble they are. This would lead us to conclude that these flours are rich in this type of compound. Calcium and reducing sugars (glucose, fructose, maltose) are low molecular weight molecules naturally soluble in water. This would mean that their high proportion in a matrix would make it more soluble.

Another grouping is phosphorus. Although not grouped to any other molecule, it is very important in the water intake of flours, the swelling power, and the ability of amylases to hydrolyze starch.

The fourth grouping is the starch, WAC, Na, SwP, AMYP, AM, carbohydrates, and WSPF. From this grouping, it appears that SwP, which is similar to water retention capacity, is strongly correlated to carbohydrates (*r* = 0.803), AMYP (*r* = 0.987), AM (*r* = −0.987), and water absorption capacity (*r* = −0.833). Carbohydrates are the main molecules responsible for water retention in flours, thanks in particular to the existence of their −OH groups. Indeed, the carbohydrate content of a food would mean less fat and protein and therefore fewer compounds preventing water retention by the starch flour and their swelling. Absorption capacity is the ability of flours to retain water and trap it. This imprisonment would therefore lead to a water stress condition in the environment, thus reducing the availability of water necessary for swelling. Amylose and amylopectin are the two components of starch. The swelling power of flours is mainly linked to starch and more particularly to its two constituents. The instability of the flour gel formed is due to the high amylose content of the flour. Indeed, amylose molecules are more soluble than amylopectin molecules due to the size of its chain and the lack of steric clutter on this molecule. It will therefore tend to dissolve in water, causing the bonds formed with water to break (Herrera, Aguirre, & Castaño, 2017). This rupture leads to a demotion of the flours, the fall of the swelling power, and the viscosity of the flours. Therefore, a flour rich in amylose will swell less. There is also a correlation between water absorption capacity and AMYP and AM content (*r* = −0.802 and 0.802, respectively). Starch and particularly amylose and amylopectin are the main molecules responsible for the rheological and functional properties of flours. In particular, it is responsible for the water retention and absorption capacity of the flours. This explains their relationship with water absorption capacity. The negative correlation between amylopectin and water absorption capacity is due to the fact that this molecule is sterically hindered and these intramolecular −OH groups have difficulty establishing bonds with molecules. Moreover, this steric clutter makes this molecule compact and difficult to detach so difficult to absorb water. The variables mentioned above, although not significantly correlated to the action of white sweet potato flour, could still influence its activity. Despite this, Na was found to be positively correlated with white sweet potato action. Indeed, just like Ca, K, and Fe, this ion plays the role of enzymatic cofactor and is therefore essential to the activity of these enzymes or enzymes in white sweet potato flour.

The analysis of the classification of flours by hierarchical ascending classification made it possible to define three classes (Figure 8). The first is made from dehulled corn and commercial flours, the second from corn, and the third from cassava and roasted cassava. These graphs show that the formation of the axes is dependent on the variables. Indeed, the formation of the F1 axis is conditioned by the cassava samples; F2 by the decoupled and commercial corn samples and F3 by the corn sample. This confirms the particular characteristics of the different flours. Indeed, this grouped behavior confirms the fact that the nature of the flour and the treatment undergone by it have an impact on the composition, functional properties, and capacity of amylase‐rich flours to predigest their starches. It also shows us that the characteristics of commercial flour and that of dehulled corn have almost the same characteristics or same functional, nutritional, and physical properties.

## 4 CONCLUSION

At the end of this work, the aim was to chemically and functionally characterize the flours and to evaluate the ability of germinated yellow corn flour and white sweet potato flour to reduce the consistency of cassava flour‐based porridges, maize, and commercial, and it appears that the approximate chemical composition and physical and functional properties of the flours vary significantly from one plant to another, from one variety to another, and from one treatment to another. The gruels made from corn, commercial, and cassava flour without flour rich in amylases are low concentrations of dry matter, and the increase in its concentration leads to an increase in consistency. The addition of flours rich in amylases during cooking resulted in a fluidification, an increase in the nutritional value, and energy density of the gruels. This reduction proved to be better with germinated maize flour where with 1%, flow rates of between 100 and 160 mm/30 s were obtained for 21.50 g DM of bitter cassava flour, thereby multiplying the energy density and nutritional value of this infant flour by 5.18. It also emerges from these analyses that the action of these amylase‐rich flours is conditioned by the nutrient composition and physical and functional properties of the flours. As well as the nutritional composition, the functional and physical properties depend on the nature of the flour and the treatment applied as shown in the PCA. In view of these results, we can therefore consider the formulation of a weaning food with a consistency, and energy and nutritional value necessary for the proper growth of children.

## CONFLICT OF INTEREST

The authors do not have any conflicting interests.

## ETHICAL REVIEW

This study does not involve any human or animal testing.

## INFORMED CONSENT

None.
